# Evaluating the 2023 safety-net kidney policy: Eligibility and outcomes of heart transplant recipients in the United States

**DOI:** 10.1016/j.xjon.2026.102087

**Published:** 2026-09-21

**Authors:** Hedwig Zappacosta, Malini Daniel, Natalie Tan, Peter D. Cho, Joseph Song, John P. White, Abbas Ardehali

**Affiliations:** a David Geffen School of Medicine, University of California, Los Angeles, Calif; b Division of Cardiac Surgery, Department of Surgery, University of California, Los Angeles, Calif; c Drexel College of Medicine, Philadelphia, Pa

**Keywords:** heart transplantation, simultaneous heart-kidney transplantation, safety-net, policy

## Abstract

**Objectives:**

In 2023, a safety-net kidney policy granting priority kidney allocation to heart transplant (HT) recipients with persistent renal dysfunction was implemented. We aim to compare early outcomes of safety-net kidney-eligible HT recipients and simultaneous heart-kidney transplant (SHKT) recipients.

**Methods:**

Adults in the United Network for Organ Sharing database between July 2023 and November 2025 were stratified into isolated heart or SHKT groups. Isolated HT recipients were further categorized by safety-net kidney eligibility, as defined by the Organ Procurement and Transplantation Network Policy. The primary outcome was 1-year survival for safety-net kidney-eligible and simultaneous heart-kidney recipients. Secondary outcomes included 6-month mortality and acute cardiac rejection within 1 year.

**Results:**

During the study period, there were 309 SHKT recipients and 181 isolated HT recipients who were safety-net kidney-eligible, 67 of whom received a kidney transplant within the study period. One-year survival was similar for safety-net kidney-eligible and SHKT recipients (96.7% vs 93.5%, *P* = .46). Mortality at 6 months did not differ between groups (2.2% vs 3.2%, *P* = .51). Acute cardiac rejection within 1 year was higher among safety-net kidney-eligible recipients (17.4% vs 8.1%, *P* = .01).

**Conclusions:**

The safety-net kidney pathway yields comparable 1-year survival compared with SHKT. However, in this early analysis, staged transplantation is associated with higher rates of acute cardiac rejection. Further studies are needed to validate the incidence and persistence of acute cardiac rejection and ascertain longer-term outcomes to better inform timing of renal transplantation for HT recipients.

**Figure:**
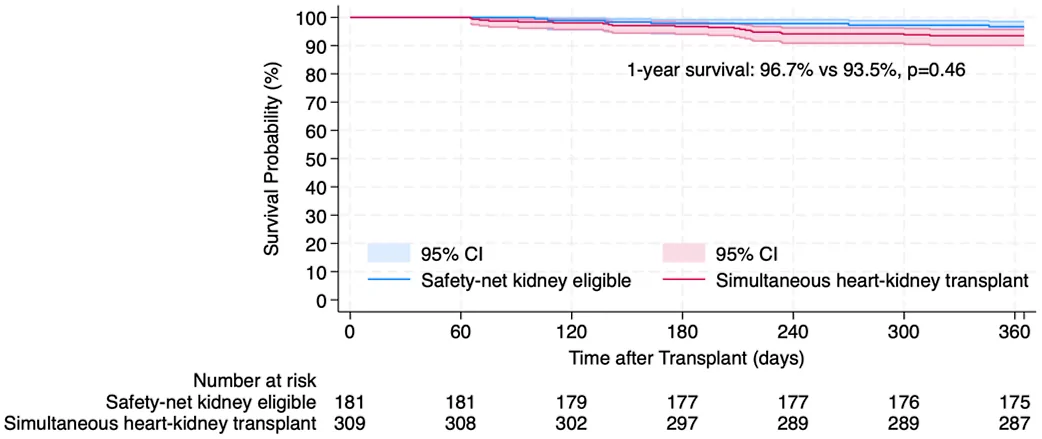
One-year survival is similar for safety-net kidney-eligible and simultaneous heart-kidney transplant recipients.

**Central Message**

Recipients of safety-net kidney transplantation after heart transplant achieve comparable 1-year survival compared with simultaneous heart-kidney transplant recipients.

**Perspective**

The safety-net kidney policy provides heart transplant recipients with a safe, effective, and accessible pathway to renal rescue, encouraging its continued and expanded use, although future studies should focus on the long-term impact of acute cardiac rejection among heart transplant recipients.

Renal dysfunction in the perioperative and short-term period following heart transplantation (HT) is a known determinant of 1-year survival.^1^, ^2^, ^3^ However, the trajectory of renal function in HT candidates is a poorly understood topic given the difficulty in ascertaining the contribution and potential reversibility of cardiorenal syndrome.^4^, ^5^, ^6^, ^7^ Furthermore, HT recipients with a postoperative need for dialysis experience significant mortality in the first weeks to months after transplant, and access to kidney transplant through the traditional kidney waitlist is associated with an average wait time of 3 to 5 years.^2^^,^^3^^,^^8^ Considering these issues, simultaneous heart-kidney transplant (SHKT) has been the historically preferred approach, even with borderline pretransplant renal dysfunction.^9^^,^^10^ However, this practice has been brought into question in recent years. There is evidence that kidney graft survival in the first year suffers from SHKT when compared to isolated HT followed by renal transplant.^11^ Furthermore, there are ethical concerns related to donor kidney allocation to SHKT recipients, who are often prioritized over isolated kidney transplant candidates.^12^

To address these concerns, the Organ Procurement and Transplantation Network (OPTN) formalized an alternative strategy via a safety-net kidney transplant policy in June 2023.^10^^,^^13^ This followed a similar and successful safety-net kidney policy for liver transplant recipients, implemented in 2017.^14^ By granting priority kidney allocation to isolated HT recipients with persistent renal failure, the policy aimed to encourage isolated HT in patients with suspected recoverable cardiorenal syndrome while providing a “rescue” pathway for those whose renal function did not recover. By providing this avenue, isolated HT may be preferred over SHKT due to the downstream safety of access to renal transplant.

Following its implementation, the real-world utilization and safety of the kidney-after-HT under the safety-net policy remain undefined. Retrospective data suggest that meeting safety-net kidney eligibility criteria is a marker for poor postoperative outcomes such as 1-year mortality.^15^ A more recent post–policy implementation study by Kwon and colleagues suggests inferior 1-year survival for safety-net kidney-eligible recipients compared to safety-net kidney noneligible recipients and SHKT recipients. However, this study maximized sensitivity for capturing safety-net kidney eligibility by including all patients who received posttransplant dialysis, regardless of renal recovery. There is no literature on the clinical outcomes for safety-net kidney-eligible isolated HT recipients, as defined by the OPTN.

Our study aims to evaluate the current safety-net policy by applying strict definitions for safety-net kidney eligibility in the post–policy era. We describe utilization and outcomes for strictly safety-net kidney-eligible recipients compared to SHKT recipients.

## Methods

### Study Population and Outcomes

All adult patients undergoing HT between July 1, 2023, and November 30, 2025, in the United Network for Organ Sharing (UNOS) database were retrospectively reviewed. Recipients undergoing redo HT or multiorgan transplant other than a heart-kidney were excluded. Recipients who had undergone a prior kidney transplant, were missing perioperative renal function data, or did not have a full year of follow-up were also excluded. Given that recipients must survive at least 60 days to meet safety-net kidney eligibility criteria, recipients who died or were lost to follow-up within 60 days after HT were excluded from the primary analysis. This study was considered exempt from review and from the need for informed written consent by the institutional review board at the University of California, Los Angeles.

Recipients were stratified into either isolated HT or SHKT cohorts. Isolated HT recipients were further categorized as safety-net kidney-eligible or noneligible groups. Safety-net kidney eligibility was defined per the OPTN definition as dialysis requirement, estimated glomerular filtration rate ≤20 mL/min, or creatinine clearance ≤20 mL/min within 60 to 365 days post-HT.^13^ Outcomes were primarily compared between safety-net kidney-eligible and SHKT cohorts, including a subanalysis on safety-net kidney-eligible recipients who received a kidney transplant during the study period. End points were analyzed in relation to recipients’ HT date. The primary end point was the 1-year survival. Secondary end points included the 6-month mortality and acute cardiac rejection within 1 year. A final subanalysis assessed the 1-year survival for safety-net kidney-eligible and SHKT recipients compared to isolated HT recipients with postoperative renal dysfunction, defined as a postoperative dialysis requirement, who never became safety-net kidney-eligible.

### Statistical Analyses

Recipient and donor characteristics were compared across groups using chi-square or Fisher exact tests on categorical variables and 2-sample *t* tests on continuous variables. Categorical variables are reported as frequencies and percentages; continuous variables are reported as medians and interquartile ranges.

To identify predictors of safety-net kidney eligibility, a multivariable logistic regression model was conducted. Clinically relevant covariates were included, and further variable selection was refined using the least absolute shrinkage and selection operator. Postoperative and early outcomes were analyzed using the chi-square or Fisher exact tests and 2-sample *t* tests. The Kaplan-Meier method was used to analyze 6-month and 1-year survival from the date of HT. To assess statistical significance for survival differences, a log-rank test was used.

To adjust for baseline differences between safety-net kidney-eligible and SHKT recipients, nearest-neighbor propensity score matching was performed at a 1:1 ratio without replacement. Variables included in the matching model were selected based on standardized mean differences (SMD) greater than 0.10 between groups prior to matching, with additional covariates retained on the grounds of clinical relevance. Balance after matching was assessed using SMD, with SMD under 0.10 considered acceptable.

In all statistical analyses, a 2-tailed *P* value below .05 was considered significant. All analyses were performed with Stata/SE 19.5 (StataCorp). This study was considered exempt from review by the institutional review board at the University of California, Los Angeles.

### Definitions

Heart size mismatch was assessed using predicted heart mass, with mismatch defined as the donor-recipient difference ≥20%.^16^ In accordance with the 2014 International Society for Heart and Lung Transplantation consensus criteria, we defined severe primary graft dysfunction (PGD) as left or biventricular dysfunction within 24 hours after transplant, not attributable to secondary causes, which required mechanical circulatory support with extracorporeal membrane oxygenation and/or a ventricular assist device.^17^

## Results

### Study Population

During the study period, 3841 HT recipients met the inclusion criteria (Figure 1). Among these, 8.9% (n = 343) underwent SHKT, of which 90.1% (n = 309) survived beyond 60 days. Of 3498 isolated HT recipients, 92.3% (n = 3228) survived beyond 60 days, and 5.6% (n = 181) of these ultimately became safety-net kidney-eligible. Among safety-net kidney-eligible recipients, 37.0% (n = 67) subsequently underwent kidney transplant during the study period, with a median time to kidney since the date of HT of 378 days (interquartile range, 282-469 days) and a median kidney waiting list time of 76 days (interquartile range, 24-189 days).

**Figure 1:**
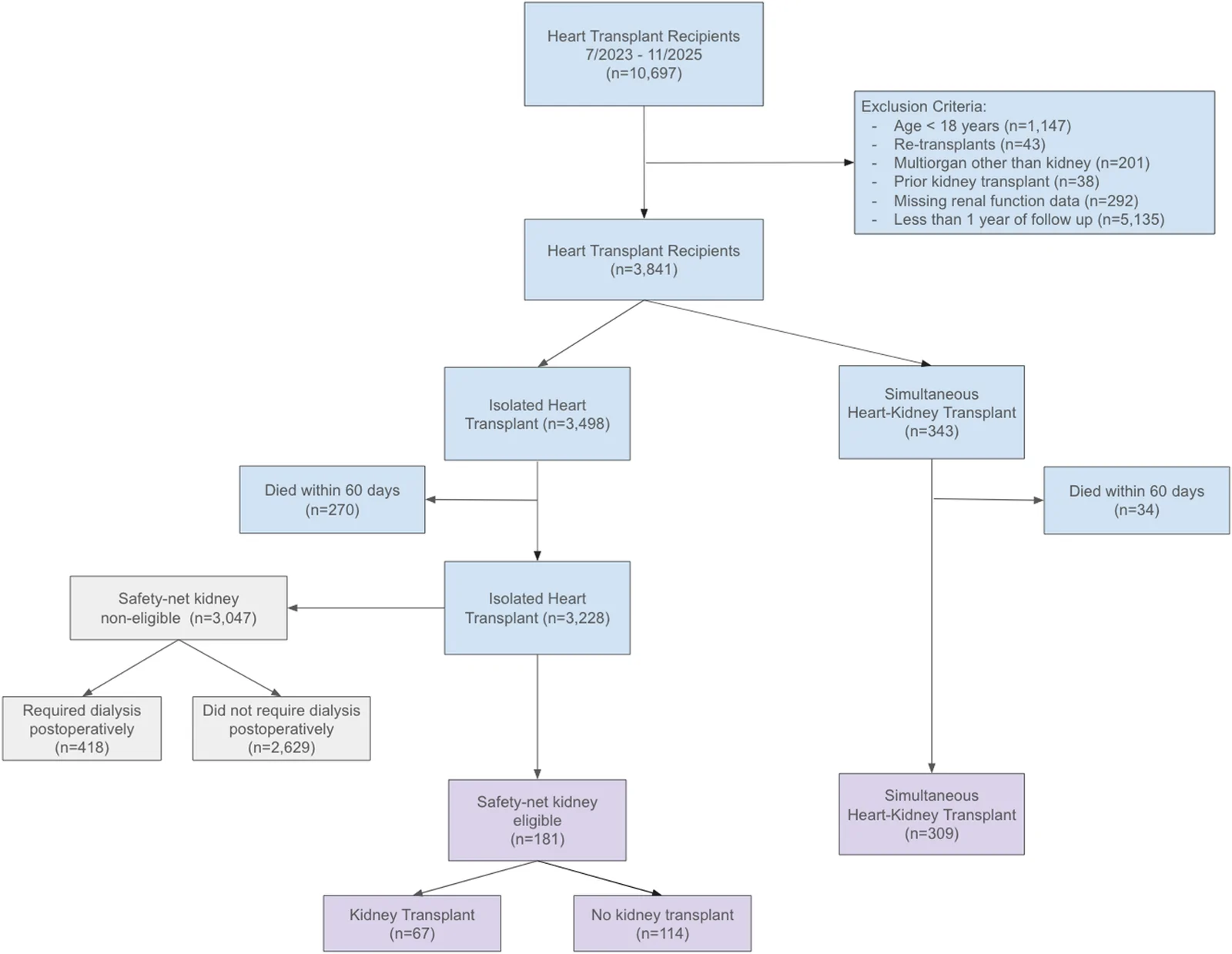
Consolidated standards of reporting trials diagram.

### Predictors of Safety-Net Kidney Eligibility

Older recipients (adjusted odds ratio [AOR], 1.02 per 1 year; *P* = .02), higher body mass index (AOR, 1.06 per 1 kg/m^2^, *P* = .01), creatinine over 1.5 mg/dL or 2.5 mg/dL during transplant (AOR, 2.40, *P* < .001; AOR, 16.47, *P* < .001), and cross-clamp-to-cross-clamp time (AOR, 1.16 per 1 hour, *P* = .01) were predictors of safety-net kidney eligibility (Table 1). Table E1 demonstrates baseline characteristics for isolated HT recipients and donors stratified by safety-net kidney eligibility. Postoperative outcomes for safety-net kidney-eligible and safety-net kidney noneligible recipients are shown in Table E2, with severe PGD (12.7% vs 3.6%, *P* < .001) and de novo dialysis after HT (55.2% vs 12.7%, *P* < .001) more common among the safety-net kidney-eligible group.

**Table 1:** Multivariable logistic regression for independent predictors of safety-net kidney eligibility among isolated heart transplant recipients

Recipient/donor characteristics	Adjusted odds ratio	95% CI	*P* value
Age, per 1 y	1.02	1.00-1.04	.02
Female gender	1.36	0.87-2.13	.17
Race			
Black	1.07	0.67-1.71	.78
Hispanic	0.97	0.48-1.97	.94
Asian	1.45	0.48-4.41	.51
Other	0.69	0.16-2.95	.61
BMI, per 1 kg/m^2^	1.06	1.02-1.10	.01
Creatinine (ref ≤1.5 mg/dL)			
1.5-2.5 mg/dL	2.40	1.51-3.81	<.001
>2.5 mg/dL	16.47	5.75-47.23	<.001
Primary diagnosis (ref NICM)	1.05	0.96-1.15	.33
Ischemic cardiomyopathy	0.55	0.32-0.93	.03
Congenital heart defect	1.75	0.63-4.89	.28
Restrictive cardiomyopathy	1.12	0.46-2.75	.80
Hypertrophic cardiomyopathy	0.82	0.28-2.42	.72
Other	0.56	0.19-1.65	.29
Inotrope use	0.86	0.56-1.33	.50
Antiarrhythmic use	1.29	0.86-1.93	.21
Dialysis	0.66	0.21-2.09	.48
Prior cardiac surgery	1.24	0.78-1.97	.37
ECMO	1.84	0.99-3.45	.06
LVAD	1.39	0.76-2.55	.29
tVAD	1.12	0.66-1.88	.68
IABP	0.72	0.28-1.34	.30
Donor pH <7.35	1.22	0.72-2.08	.46
Undersized donor heart	0.78	0.31-1.95	.60
Cross-clamp-to-cross-clamp time >4 h	1.16	1.04-1.29	.01

*BMI*, Body mass index; *ECMO*, extracorporeal membrane oxygenation; *IABP*, intra-aortic balloon pump; *LVAD*, left ventricular assist device; *NICM*, non–ischemic dilated cardiomyopathy; *tVAD*, temporary ventricular assist device.

### Safety-Net Kidney-Eligible Versus SHKT: Unmatched Analyses

Compared to SHKT recipients, safety-net kidney-eligible recipients were less likely to require dialysis prior to transplant (7.7% vs 41.7%, *P* < .001) but more likely to have a history of prior cardiac surgery (43.6% vs 29.8%, *P* = .01), left ventricular assist device requirement (21.0% vs 10.4%, *P* = .01), or extracorporeal membrane oxygenation requirement (12.2% vs 6.5%, *P* = .03) (Table 2). Heart donor characteristics are shown in Table E3. The safety-net kidney-eligible cohort demonstrated comparable 1-year survival compared to SHKT recipients (96.7% vs 93.5%, *P* = .46) (Figure 2, *A*). There were no deaths observed among safety-net kidney-eligible recipients who underwent kidney transplant (100.0% vs 93.5%, *P* = .17) (Figure 2, *B*). Six-month mortality was similar across groups (2.2% vs 3.2%, *P* = .51) (Table 3). However, safety-net kidney-eligible recipients were more likely to be treated for acute cardiac rejection within 1 year of HT (17.4% vs 8.1%, *P* = .01). Higher rates of acute cardiac rejection within 1 year were also observed among safety-net kidney-eligible recipients who received a kidney transplant compared with SHKT recipients, though results did not reach statistical significance (13.6% vs 8.1%, *P* = .16).

**Table 2:** Baseline characteristics of safety-net kidney-eligible versus SHKT recipients

Recipient characteristics	Safety-net kidney-eligible	SHKT	*P* value
	N = 181	N = 309	
Age, y	56 (48-64)	59 (52-64)	.12
Gender			.87
Male	133 (73.5%)	225 (72.8%)	
Female	48 (26.5%)	84 (27.2%)	
Race			.08
White	100 (55.2%)	133 (43.0%)	
Black	61 (33.7%)	123 (39.8%)	
Hispanic	14 (7.7%)	32 (10.4%)	
Asian	4 (2.2%)	16 (5.2%)	
Other	2 (1.1%)	5 (1.6%)	
Smoking history	66 (36.5%)	118 (38.2%)	.70
Diabetes mellitus	65 (35.9%)	159 (51.5%)	<.001
Prior cardiac surgery	79 (43.6%)	92 (29.8%)	.01
BMI, kg/m^2^	29.7 (25.5-33.9)	27.6 (24.5-31.3)	<.001
Functional status (1-10)	3 (2-7)	4 (2-6)	.45
Creatinine			<.001
≤1.5 mg/dL	109 (60.2%)	33 (10.7%)	
1.5-2.5 mg/dL	57 (31.5%)	121 (39.2%)	
≥2.5 mg/dL	15 (8.3%)	155 (50.2%)	
Total bilirubin, mg/dL	0.6 (0.4-0.9)	0.6 (0.4-1.0)	.93
Pulmonary hypertension	116 (64.1%)	247 (79.9%)	<.001
Cardiac output, L/min	4.5 (3.71-5.61)	4.55 (3.6-5.7)	.69
Primary diagnosis			.01
Non–ischemic dilated cardiomyopathy	108 (59.7%)	164 (53.1%)	
Ischemic cardiomyopathy	37 (20.4%)	108 (35.0%)	
Congenital heart defect	11 (6.1%)	4 (1.3%)	
Restrictive cardiomyopathy	10 (5.5%)	11 (3.6%)	
Valvular heart disease	0 (0.0%)	2 (0.6%)	
Hypertrophic cardiomyopathy	6 (3.3%)	8 (2.6%)	
Other	9 (5.0%)	12 (3.9%)	
Admission status			.37
ICU	107 (59.1%)	202 (65.4%)	
Hospitalized, non-ICU	36 (19.9%)	54 (17.5%)	
Not hospitalized	38 (21.0%)	53 (17.2%)	
Dialysis prior to transplant	14 (7.7%)	129 (41.7%)	<.001
Mechanical ventilation	5 (2.8%)	2 (0.6%)	.06
LVAD	38 (21.0%)	32 (10.4%)	.01
tVAD	50 (27.6%)	86 (27.8%)	.96
IABP	28 (15.5%)	60 (19.4%)	.27
ECMO	22 (12.2%)	20 (6.5%)	.03
Status 1	40 (22.1%)	43 (13.9%)	.02
Status 2	89 (49.2%)	175 (56.6%)	.11
Status 3-6	52 (28.7%)	91 (29.4%)	.87
Days on waiting list	21 (11-60)	38 (15-125)	<.001

Categorical values are reported as frequency (% of population). Continuous values are reported as median (IQR). *BMI*, Body mass index; *ECMO*, extracorporeal membrane oxygenation; *IABP*, intra-aortic balloon pump; *ICU*, intensive care unit; *LVAD*, left ventricular assist device; *SHKT*, simultaneous heart-kidney transplant; *tVAD*, temporary ventricular assist device; *IQR*, interquartile range.

**Figure 2:**
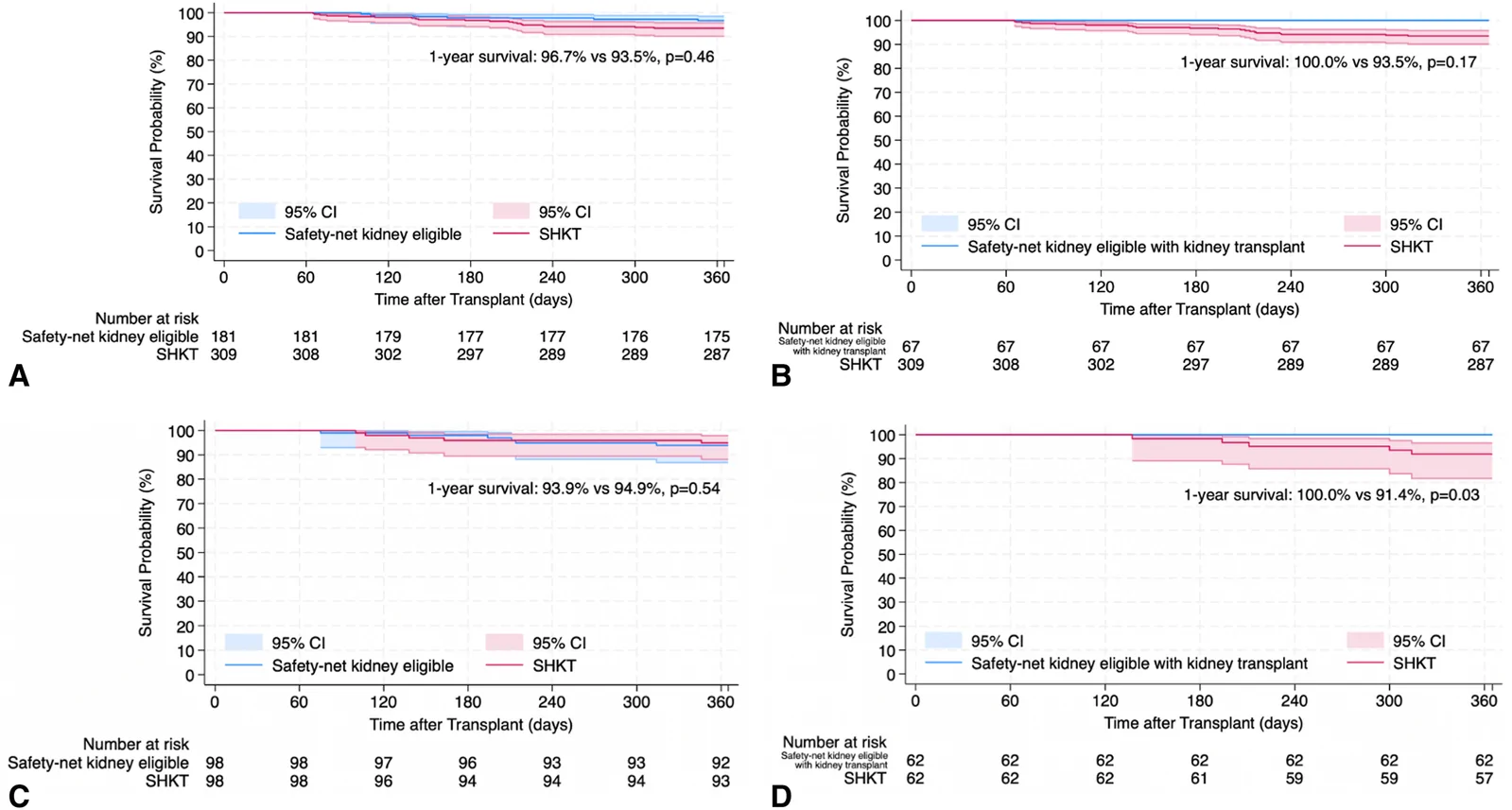
One-year Kaplan-Meier survival analysis for (A) safety-net kidney-eligible versus SHKT recipients, (B) safety-net kidney-eligible with kidney transplant versus SHKT recipients, (C) matched safety-net kidney-eligible versus SHKT recipients, and (D) matched safety-net kidney-eligible with kidney transplant versus SHKT recipients. *SHKT*, simultaneous heart-kidney transplant.

**Table 3:** Short-term outcomes of safety-net kidney-eligible versus SHKT recipients

Endpoints	Safety-net kidney-eligible^a^ N = 181	Safety-net kidney-eligible with kidney transplant^b^ N = 67	SHKT^c^ N = 309	*P* value (a vs c)	*P* value (b vs c)
6-month mortality	4 (2.2%)	0 (0.0%)	10 (3.2%)	.51	.14
Acute cardiac rejection within 1 year	30 (17.4%)	9 (13.6%)	23 (8.1%)	.01	.16

Values are reported as frequency (% of population). a, b, and c identify the second, third, and fourth columns, respectively, and *P* values indicate the significance of the comparisons between the corresponding columns. *SHKT*, Simultaneous heart-kidney transplant.

### Safety-Net Kidney-Eligible Versus SHKT: Matched Analyses

Propensity score matching yielded 98 well-balanced SHKT/safety-net kidney-eligible pairs and yielded 62 well-balanced pairs for SHKT/safety-net kidney-eligible recipients who received a kidney transplant (Tables E4 and E5). One-year survival did not differ significantly between matched safety-net kidney-eligible and SHKT recipients (93.9% vs 94.9%, *P* = .54) (Figure 2, *C*), and no deaths were observed among safety-net kidney-eligible recipients who underwent kidney transplant (100.0% vs 91.4%, *P* = .03) (Figure 2, *D*). Acute cardiac rejection within 1 year was more prevalent among matched safety-net kidney-eligible recipients compared to SHKT recipients and among matched safety-net kidney-eligible recipients who received a kidney transplant compared to SHKT recipients, though results did not reach statistical significance (17.3% vs 9.2%, *P* = .09; 14.5% vs 8.1%, *P* = .28) (Table 4).

**Table 4:** Short-term outcomes of matched safety-net kidney-eligible versus SHKT recipients

Endpoints	Safety-net kidney-eligible N = 98	SHKT N = 98	*P* value	Safety-net kidney-eligible with kidney transplant N = 62	SHKT N = 62	*P* value
6-mo mortality	4 (4.1%)	2 (2.0%)	.41	0 (0.0%)	0 (0.0%)	.32
Acute cardiac rejection within 1 y	17 (17.3%)	9 (9.2%)	.09	9 (14.5%)	5 (8.1%)	.28

Values are reported as frequency (% of population); *SHKT*, Simultaneous heart-kidney transplant.

### Outcomes of Isolated HT Recipients With Postoperative Renal Dysfunction Who Did Not Become Safety-Net Kidney-Eligible

Recipient and heart donor characteristics for safety-net kidney-eligible and safety-net kidney noneligible HT recipients who required postoperative dialysis are shown in Table E6. Survival at 1-year post-HT was lowest among safety-net kidney noneligible isolated HT recipients who required postoperative dialysis when compared to safety-net kidney-eligible and SHKT recipients (81.3% vs 96.7% vs 93.5%, global *P* < .001) (Figure 3). A subanalysis of 60-day survival for these groups is shown in Figure E1. Causes of death did not significantly differ between safety-net kidney noneligible HT recipients who required postoperative dialysis compared to safety-net kidney-eligible recipients (Table E7).

**Figure 3:**
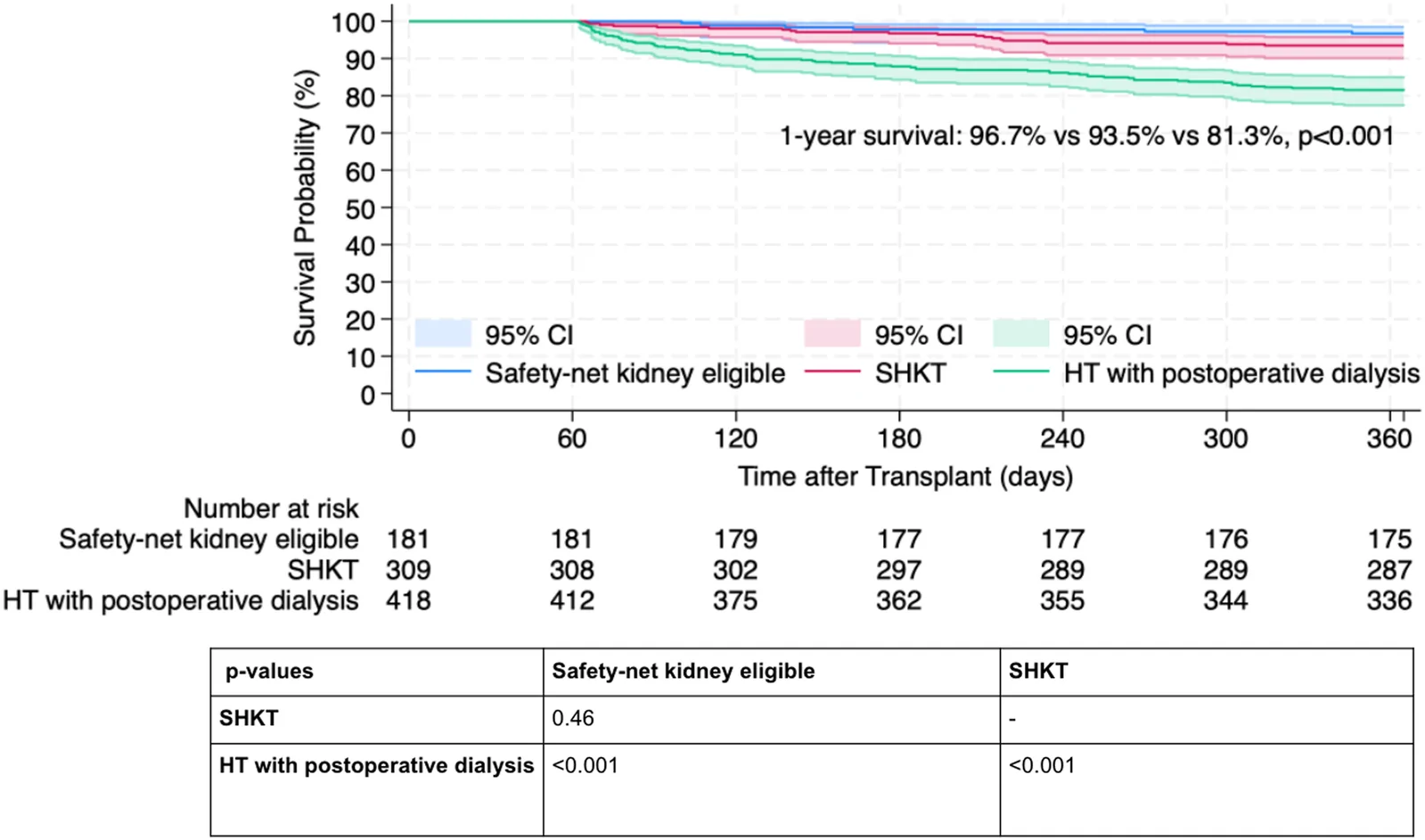
One-year Kaplan-Meier survival analysis of isolated HT recipients who required postoperative dialysis compared to safety-net kidney-eligible and SHKT recipients. *HT*, Heart transplant; *SHKT*, simultaneous heart-kidney transplant.

## Discussion

This study represents the first real-world national outcomes analysis for HT recipients following the safety-net kidney policy implementation in 2023. We observe comparable short-term survival for SHKT and kidney-after-heart recipients under the current policy, encouraging isolated HT over upfront combined organ transplant when clinically indicated. However, safety-net kidney-eligible recipients exhibited a trend toward higher rates of acute cardiac rejection within 1 year of HT compared to SHKT recipients. Considering this, our study supports the short-term safety of the safety-net kidney pathway while necessitating continued monitoring for acute rejection and longer-term outcomes.

The safety of kidney-after-HT under the safety-net policy compared to upfront SHKT is supported by comparable survival across these groups up to 1-year post-HT. This finding, together with the accessibility of renal transplant provided by the safety-net pathway, supports the feasibility of kidney-after-heart as a safe alternative to SHKT in the short term. Notably, on initial evaluation, our results seem to conflict with Kwon and colleagues’ findings that 1-year survival is inferior among safety-net kidney-eligible recipients compared to SHKT.^18^ However, due to the early nature of their analysis, Kwon’s group chose to focus on sensitivity, capturing the spectrum of patients requiring postoperative dialysis rather than focusing only on the patients that were safety-net kidney-eligible. With a longer follow-up period, the strength of our study lies in our ability to apply a strict definition of safety-net kidney eligibility criteria outlined by the OPTN. We thereby provide the first report of the true safety for the safety-net kidney policy in the modern era. With time, the trend should favor specificity for selecting safety-net kidney-eligible cohorts for further analysis of this policy.

While we have noted similar 1-year survival across safety-net kidney-eligible and SHKT recipients, our analyses also reveal a potentially higher rate of acute cardiac rejection within 1 year among safety-net kidney-eligible recipients. This finding may be explained by the receipt of multiple organs from 1 donor with SHKT, a pattern previously observed in the literature.^19^ It is also plausible that the use of renal sparing immunosuppressive strategies in either SHKT recipients or safety-net kidney-eligible recipients influences acute rejection outcomes, though we lack this granular data in the UNOS database and are unable to comment on immunosuppressive strategies used in our cohorts.^5^ Furthermore, it is possible that the persistent renal dysfunction which defines the safety-net cohort may contribute to the development of acute cardiac rejection, an association noted in the literature.^2^ The mechanism behind this association remains unclear, though, as renal disease may contribute to a mixed picture of immunosuppression and immunoactivation.^20^ Although short-term survival outcomes are encouraging, this association between safety-net eligibility and acute rejection warrants further investigation, including longitudinal studies of cardiac graft function, short- and long-term incidence of cardiac allograft vasculopathy, and mid- to long-term survival.

A cohort of 418 isolated HT recipients who required postoperative dialysis did not meet safety-net kidney eligibility criteria and experienced substantially worse survival at 1 year compared to safety-net kidney-eligible and SHKT recipients. Importantly, this cohort contains at least 2 subgroups: those whose renal function recovered and those who were simply too sick to reasonably be listed for a second transplant. Unfortunately, the UNOS database does not provide adequate granularity to differentiate among these groups. Further multicenter or single-institution studies on the second subgroup would lend critical insight into organ utilization and allocation. Acknowledging the significant mortality of these HT recipients raises important questions about which patients the safety-net kidney policy captures and about heart allocation to this high-risk group. Nonetheless, it is encouraging that some isolated HT recipients who require postoperative dialysis will eventually recover renal function and do not require rescue kidney transplantation.

Several limitations of this study are acknowledged. As with all large database analyses, results are subject to potential data entry inaccuracies and lack of clinical granularity. Notably, the trajectory of recipient renal function cannot be clearly depicted through the UNOS database, as serial renal function data such as creatinine clearance and estimated glomerular filtration rate are not available for those not on the kidney waitlist. Furthermore, although we conducted a propensity matched analysis, unmeasurable differences between our safety-net kidney-eligible and SHKT cohorts likely still exist, such as nuances of key clinician or center-specific decision-making regarding patient candidacy for SHKT at the time of organ offer. Most importantly, recipients who died or were lost to follow-up within 60 days after HT were excluded from our core analysis, presenting an inherent survivor bias. Although we cannot predict which isolated HT recipients would have become safety-net kidney-eligible had they survived beyond 60 days, an analysis of 60-day mortality for isolated HT recipients with postoperative dialysis requirement may give some insight into the extent of bias introduced. SHKT recipients experienced a 10.3% 60-day mortality rate, while HT recipients with postoperative dialysis requirement experienced a 19.7% 60-day mortality rate. Notably, the latter number may be overestimating mortality, as those with renal dysfunction not requiring postoperative dialysis are not included due to limitations of the UNOS database.

## Conclusions

Overall, our analyses suggest that the safety-net kidney pathway is functioning to preserve short-term survival among HT recipients with persistent postoperative renal dysfunction, demonstrating comparable survival to SHKT recipients. However, utilization of the safety-net kidney pathway may increase rates of acute rejection for the cardiac graft when compared to SHKT. As experience with the policy develops, future studies should focus on graft function, cardiac allograft vasculopathy incidence, and mid- to long-term survival for safety-net kidney-eligible recipients to characterize the durability of renal outcomes and clarify the role of acute cardiac rejection as a long-term risk in this population. Nonetheless, our findings support an early strategy that preferences staged organ transplant over SHKT when clinically indicated, such as in cases of borderline pre-HT renal dysfunction or in cases of post-HT hemodynamic instability when simultaneous kidney transplant may not be preferred.

### Webcast

You can watch a Webcast of this AATS meeting presentation by going to: https://www.aats.org/resources/kidney-after-heart-versus-simu-12264.

**Figure:**
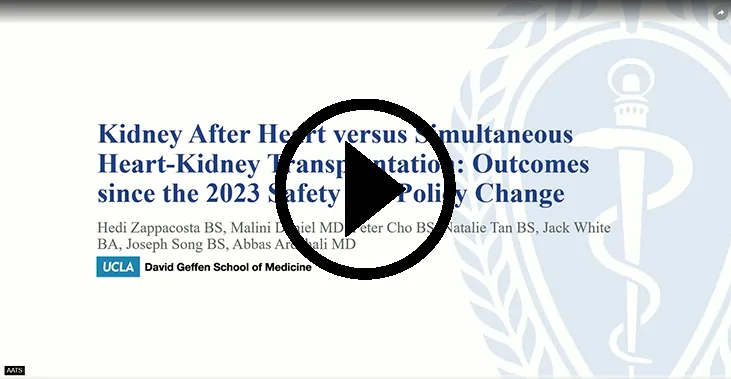

## Conflict of Interest Statement

The authors reported no conflicts of interest.

The *Journal* policy requires editors and reviewers to disclose conflicts of interest and to decline handling or reviewing manuscripts for which they may have a conflict of interest. The editors and reviewers of this article have no conflicts of interest.

## Figures and Tables

**Table E1:** Baseline characteristics of isolated heart transplant recipients and donors stratified by safety-net kidney eligibility

Recipient/donor characteristics	Safety-net kidney-eligible	Safety-net kidney noneligible	*P* value
	N = 181	N = 3047	
Recipients			
Age, y	56 (48-64)	57 (47-64)	.47
Gender			.84
Male	133 (73.5%)	2218 (72.8%)	
Female	48 (26.5%)	829 (27.2%)	
Race			.04
White	100 (55.2%)	1730 (56.8%)	
Black	61 (33.7%)	762 (25.0%)	
Hispanic	14 (7.7%)	371 (12.2%)	
Asian	4 (2.2%)	102 (3.3%)	
Other	2 (1.1%)	82 (2.7%)	
Smoking history	66 (36.5%)	1211 (39.7%)	.38
Diabetes mellitus	65 (35.9%)	910 (29.9%)	.09
Prior cardiac surgery	79 (43.6%)	985 (32.3%)	.01
BMI, kg/m^2^	29.7 (25.5-33.9)	27.5 (24.1-31.2)	<.001
Functional status (1-10)	3 (2-7)	4 (2-7)	.48
Creatinine			<.001
≤1.5 mg/dL	109 (60.2%)	2583 (84.8%)	
1.5-2.5 mg/dL	57 (31.5%)	436 (14.3%)	
≥2.5 mg/dL	15 (8.3%)	28 (0.9%)	
Total bilirubin, mg/dL	0.6 (0.4-0.9)	0.7 (0.4-1.0)	.21
Pulmonary hypertension	116 (64.1%)	1844 (60.5%)	.34
Cardiac output, L/min	4.5 (3.7-5.6)	4.21 (3.4-5.2)	.01
Primary diagnosis			.17
Non–ischemic dilated cardiomyopathy	108 (59.7%)	1655 (54.3%)	
Ischemic cardiomyopathy	37 (20.4%)	832 (27.3%)	
Congenital heart defect	11 (6.1%)	113 (3.7%)	
Restrictive cardiomyopathy	10 (5.5%)	142 (4.7%)	
Valvular heart disease	0 (0.0%)	35 (1.1%)	
Hypertrophic cardiomyopathy	6 (3.3%)	124 (4.1%)	
Other	9 (5.0%)	146 (4.8%)	
Admission status			.15
ICU	107 (59.1%)	1945 (63.8%)	
Hospitalized, non-ICU	36 (19.9%)	447 (14.7%)	
Not hospitalized	38 (21.0%)	655 (21.5%)	
Dialysis prior to transplant	14 (7.7%)	65 (2.1%)	<.001
Mechanical ventilation	5 (2.8%)	75 (2.5%)	.80
LVAD	38 (21.0%)	484 (15.9%)	.07
tVAD	50 (27.6%)	837 (27.5%)	.96
IABP	28 (15.5%)	634 (20.8%)	.08
ECMO	22 (12.2%)	251 (8.2%)	.07
Status 1	40 (22.1%)	438 (14.4%)	.01
Status 2	89 (49.2%)	1693 (55.6%)	.09
Status 3-6	52 (28.7%)	896 (29.4%)	.85
Days on waiting list	21 (11-60)	25 (9-80)	.48
Donors			
Age, y	37 (28-44)	34 (26-41)	.01
Gender			.72
Male	129 (71.3%)	2134 (70.0%)	
Female	52 (28.7%)	913 (30.0%)	
Race			.84
White	109 (60.2%)	1890 (62.0%)	
Black	31 (17.1%)	483 (15.9%)	
Hispanic	33 (18.2%)	558 (18.3%)	
Asian	3 (1.7%)	63 (2.1%)	
Other	5 (2.8%)	53 (1.7%)	
Smoking history	31 (17.1%)	433 (14.2%)	.28
Hypertension	33 (18.2%)	554 (18.2%)	.99
Cause of death			.45
Anoxia	80 (44.2%)	1467 (48.1%)	
Cerebrovascular/stroke	23 (12.7%)	378 (12.4%)	
Head trauma	69 (38.1%)	1102 (36.2%)	
CNS tumor	3 (1.7%)	19 (0.6%)	
Other	6 (3.3%)	81 (2.7%)	
BMI, kg/m^2^	28.3 (23.3-33.2)	27.1 (23.6-31.4)	.06
Creatinine, mg/dL	0.9 (0.7-1.7)	0.9 (0.7-1.6)	.33
Terminal pH <7.35	19 (16.2%)	274 (13.3%)	.36
Donor-recipient match			
Undersized donor heart	11 (6.1%)	139 (4.6%)	.11
HLA mismatch level	5 (4-5)	5 (4-5)	.56
Cross-clamp-to-cross-clamp time, hours	4.0 (3.3-4.9)	3.7 (3.1-4.6)	.01

Categorical values are reported as frequency (% of population). Continuous values are reported as median (IQR). *BMI*, Body mass index; *CNS*, central nervous system; *ECMO*, extracorporeal membrane oxygenation; *HLA*, human leukocyte antigen; *HT-dialysis*, heart transplant requiring predischarge dialysis; *IABP*, intra-aortic balloon pump; *LVAD*, left ventricular assist device; *tVAD*, temporary ventricular assist device; *IQR*, interquartile range.

**Table E2:** Postoperative differences between safety-net kidney-eligible and safety-net kidney noneligible isolated heart transplant recipients

Endpoints	Safety-net kidney-eligible	Safety-net kidney noneligible	*P* value
	N = 181	N = 3047	
Severe PGD	23 (12.7%)	110 (3.6%)	<.001
Right ventricular PGD	8 (4.4%)	89 (2.9%)	.25
Predischarge dialysis			
Total	113 (62.4%)	418 (13.7%)	<.001
New dialysis after HT	100 (55.2%)	386 (12.7%)	<.001
Stroke	9 (5.0%)	120 (3.9%)	.49
Pacemaker	1 (0.6%)	48 (1.6%)	.06
30-day graft failure	0 (0.0%)	1 (0.0%)	.81
Acute rejection	29 (16.0%)	439 (14.4%)	.55

Categorical values are reported as frequency (% of population). *HT*, Heart transplantation; *PGD*, primary graft dysfunction.

**Table E3:** Baseline characteristics of safety-net kidney-eligible versus SHKT heart donors

Donor characteristics	Safety-net kidney-eligible	SHKT	*P* value
	N = 181	N = 309	
Age, y	37 (28-44)	33 (26-41)	.01
Gender			.45
Male	129 (71.3%)	230 (74.4%)	
Female	52 (28.7%)	79 (25.6%)	
Race			.43
White	109 (60.2%)	187 (60.5%)	
Black	31 (17.1%)	50 (16.2%)	
Hispanic	33 (18.2%)	66 (21.4%)	
Asian	3 (1.7%)	1 (0.3%)	
Other	5 (2.8%)	5 (1.6%)	
Smoking history	31 (17.1%)	40 (12.9%)	.20
Hypertension	33 (18.2%)	43 (13.9%)	.20
Cause of death			.61
Anoxia	80 (44.2%)	134 (43.4%)	
Cerebrovascular/stroke	23 (12.7%)	29 (9.4%)	
Head trauma	69 (38.1%)	135 (43.7%)	
CNS tumor	3 (1.7%)	3 (1.0%)	
Other	6 (3.3%)	8 (2.6%)	
BMI, kg/m^2^	28.3 (23.3-33.2)	26.9 (23.4-30.7)	.04
Creatinine, mg/dL	0.9 (0.7-1.7)	0.9 (0.7-1.1)	.52
Terminal pH <7.35	19 (16.2%)	24 (11.5%)	.23
Donor-recipient match			
Undersized donor heart	11 (6.1%)	11 (3.6%)	.17
HLA mismatch level	5 (4-5)	5 (4-6)	.53
Cross-clamp-to-cross-clamp time, h	4.0 (3.3-4.9)	3.8 (3.1-4.5)	.02

Categorical values are reported as frequency (% of population). Continuous values are reported as median (IQR). *BMI*, Body mass index; *CNS*, central nervous system; *ECMO*, extracorporeal membrane oxygenation; *HLA*, human leukocyte antigen; *IABP*, intra-aortic balloon pump; *LVAD*, left ventricular assist device; *SHKT*, simultaneous heart-kidney transplant; *tVAD*, temporary ventricular assist device; *IQR*, interquartile range.

**Table E4:** Baseline characteristics of propensity matched safety-net kidney-eligible and SHKT recipients and donors

Recipient/donor characteristics	Safety-net kidney-eligible	SHKT	*P* value	SMD
	N = 98	N = 98		
Recipients				
Age, y	58 (52-66)	59 (50-64)	.42	−0.06
Gender			.75	−0.04
Male	71 (72%)	73 (74%)		
Female	27 (28%)	25 (26%)		
Race			.61	
White	50 (51%)	48 (49%)		
Black	37 (38%)	34 (35%)		
Hispanic	8 (8%)	9 (9%)		
Asian	3 (3%)	7 (7%)		
Smoking history	34 (35%)	33 (34%)	.88	
Diabetes mellitus	39 (40%)	50 (51%)	.11	
Prior cardiac surgery	33 (34%)	37 (38%)	.55	0.09
BMI, kg/m^2^	29.5 (25.3-32.6)	27.6 (24.6-31.4)	.05	
Functional status (1-10)	3 (2-6)	3 (2-5)	.60	
Creatinine, mg/dL	1.7 (1.3-2.2)	1.8 (1.4-2.3)	.09	−0.03
Pulmonary hypertension	68 (69%)	81 (83%)	.03	
Cardiac output, L/min	4.235 (3.63-5.44)	4.65 (3.925-5.755)	.10	
Primary diagnosis			.28	
Non–ischemic dilated cardiomyopathy	56 (57%)	46 (47%)		
Ischemic cardiomyopathy	24 (24%)	36 (37%)		
Congenital heart defect	5 (5%)	3 (3%)		
Restrictive cardiomyopathy	6 (6%)	4 (4%)		
Valvular heart disease	0 (0%)	2 (2%)		
Hypertrophic cardiomyopathy	2 (2%)	4 (4%)		
Other	5 (5%)	3 (3%)		
Admission status			.65	
ICU	68 (69%)	69 (70%)		
Hospitalized, non-ICU	13 (13%)	16 (16%)		
Not hospitalized	17 (17%)	13 (13%)		
Dialysis prior to transplant	11 (11%)	35 (36%)	<.001	
Mechanical ventilation	3 (3%)	2 (2%)	.65	
LVAD	15 (15%)	16 (16%)	.84	0.03
tVAD	34 (35%)	31 (32%)	.65	
IABP	17 (17%)	18 (18%)	.85	
ECMO	10 (10%)	12 (12%)	.65	0.06
Status 1	20 (20%)	22 (22%)	.73	0.05
Status 2	54 (55%)	55 (56%)	.89	
Status 3-6	24 (24%)	21 (21%)	.61	
Days on waiting list	22.5 (11-60)	33 (12-78)	.29	−0.024
Donors				
Age, y	35 (24-43)	35 (29-41)	.94	0.02
Gender			.53	0.09
Male	72 (73%)	68 (69%)		
Female	26 (27%)	30 (31%)		0.09
Race			.49	
White	60 (61%)	69 (70%)		
Black	13 (13%)	10 (10%)		
Hispanic	21 (21%)	17 (17%)		
Asian	2 (2%)	0 (0%)		
Other	2 (2%)	2 (2%)		
Smoking history	15 (15%)	11 (11%)	.40	
Steroid use	66 (67%)	62 (63%)	.55	−0.09
Hypertension	17 (17%)	13 (13%)	.43	
Cause of death			.60	
Anoxia	46 (47%)	45 (46%)		
Cerebrovascular/stroke	9 (9%)	14 (14%)		
Head trauma	38 (39%)	37 (38%)		
CNS tumor	1 (1%)	0 (0%)		
Other	4 (4%)	2 (2%)		
BMI, kg/m^2^	28.1 (23.1-32.5)	28.4 (24.3-32.0)	.93	
Creatinine, mg/dL	0.9 (0.7-1.7)	0.9 (0.7-1.1)	.08	
Total bilirubin, mg/dL	0.7 (0.4-1.0)	0.6 (0.4-1.1)	.52	0.02
Terminal pH <7.35	9 (16%)	5 (8%)	.17	−0.04
Donor-recipient match				
Undersized donor heart	7 (7%)	3 (3%)	.27	
HLA mismatch level	5 (4-5)	5 (4-6)	.37	
Cross-clamp-to-cross-clamp time, h	3.7 (3.3-4.8)	3.8 (3.1-4.3)	.26	

Categorical values are reported as frequency (% of population). Continuous values are reported as median (IQR). *BMI*, Body mass index; *CNS*, central nervous system; *ECMO*, extracorporeal membrane oxygenation; *HLA*, human leukocyte antigen; *IABP*, intra-aortic balloon pump; *LVAD*, left ventricular assist device; *SHKT*, simultaneous heart-kidney transplant; *SMD*, standardized mean difference; *tVAD*, temporary ventricular assist device; *IQR*, interquartile range.

**Table E5:** Baseline characteristics of matched safety-net kidney-eligible with kidney transplant and simultaneous heart-kidney transplant recipients and donors

Recipient/donor characteristics	Safety-net kidney-eligible with kidney transplant	SHKT	*P* value	SMD
	N = 62	N = 62		
Recipients				
Age, y	56 (47-65)	62 (55-66)	.03	
Gender			.01	
Male	52 (84%)	40 (65%)		
Female	10 (16%)	22 (35%)		
Race			.58	
White	36 (58%)	34 (55%)		
Black	21 (34%)	21 (34%)		
Hispanic	3 (5%)	3 (5%)		
Asian	1 (2%)	4 (6%)		
Other	1 (2%)	0 (0%)		
Smoking history	18 (29%)	27 (44%)	.09	
Diabetes mellitus	24 (39%)	30 (48%)	.28	
Prior cardiac surgery	25 (40%)	25 (40%)	1.00	0.00
BMI, kg/m^2^	30.6 (26.8-34.1)	28.7 (25.3-31.9)	.08	
Functional status (1-10)	3 (2-6)	4 (2-6)	.31	
Creatinine, mg/dL	1.6 (1.4-2.1)	1.7 (1.3-2.1)	.75	0.05
Total bilirubin, mg/dL	0.7 (0.5-1.0)	0.7 (0.4-0.9)	.97	−0.05
Pulmonary hypertension	43 (69%)	50 (81%)	.15	
Cardiac output, L/min	4.7 (3.8-5.5)	4.4 (3.5-5.5)	.96	
Primary diagnosis			.11	−0.02
Non–ischemic dilated cardiomyopathy	40 (65%)	32 (52%)		
Ischemic cardiomyopathy	9 (15%)	20 (32%)		
Congenital heart defect	3 (5%)	0 (0%)		
Restrictive cardiomyopathy	4 (6%)	3 (5%)		
Valvular heart disease	0 (0%)	2 (3%)		
Hypertrophic cardiomyopathy	1 (2%)	1 (2%)		
Other	5 (8%)	4 (6%)		
Admission status			.97	−0.021
ICU	40 (65%)	39 (63%)		
Hospitalized, non-ICU	12 (19%)	13 (21%)		
Not hospitalized	10 (16%)	10 (16%)		
Dialysis prior to transplant	7 (11%)	16 (26%)	.04	
Mechanical ventilation	1 (2%)	0 (0%)	.32	
LVAD	9 (15%)	11 (18%)	.63	−0.09
tVAD	18 (29%)	20 (32%)	.70	
IABP	7 (11%)	8 (13%)	.78	
ECMO	7 (11%)	7 (11%)	1.00	0.00
Status 1	12 (19%)	14 (23%)	.66	0.08
Status 2	36 (58%)	32 (52%)	.47	
Status 3-6	14 (23%)	16 (26%)	.67	
Days on waiting list	20 (11-58)	28 (9-153)	.28	
Donors				
Age, y	38 (25-47)	32 (24-38)	.04	
Gender			.19	
Male	52 (84%)	46 (74%)		
Female	10 (16%)	16 (26%)		
Race			.29	
White	30 (48%)	40 (65%)		
Black	14 (23%)	8 (13%)		
Hispanic	16 (26%)	12 (19%)		
Asian	1 (2%)	0 (0%)		
Other	1 (2%)	2 (3%)		
Smoking history	9 (15%)	6 (10%)	.41	
Steroid use	45 (73%)	44 (71%)	.84	0.04
Hypertension	11 (18%)	4 (6%)	.05	
Cause of death			.69	
Anoxia	28 (45%)	31 (50%)		
Cerebrovascular/stroke	8 (13%)	7 (11%)		
Head trauma	22 (35%)	23 (37%)		
CNS tumor	1 (2%)	0 (0%)		
Other	3 (5%)	1 (2%)		
BMI, kg/m^2^	27.9 (23.7-32.2)	28.2 (24.4-31.7)	.79	
Creatinine, mg/dL	0.9 (0.7-1.6)	0.9 (0.7-1.1)	.10	
Terminal pH <7.35	6 (15%)	2 (5%)	.11	0.01
Donor-recipient match				
Undersized donor heart	6 (10%)	1 (2%)	.02	
HLA mismatch level	5 (4-6)	5 (4-6)	.84	
Cross-clamp-to-cross-clamp time, h	3.9 (3.3-4.9)	3.9 (3.1-4.6)	.54	0.07

Categorical values are reported as frequency (% of population). Continuous values are reported as median (IQR). *BMI*, Body mass index; *CNS*, central nervous system; *ECMO*, extracorporeal membrane oxygenation; *HLA*, human leukocyte antigen; *IABP*, intra-aortic balloon pump; *LVAD*, left ventricular assist device; *SHKT*, simultaneous heart-kidney transplant; *SMD*, standardized mean difference; *tVAD*, temporary ventricular assist device; *IQR*, interquartile range.

**Table E6:** Recipient and heart donor characteristics of safety-net kidney-eligible and safety-net kidney noneligible HT recipients who required postoperative dialysis

Recipient/donor characteristics	Safety-net kidney-eligible	Isolated HT with postoperative dialysis	*P* value
	N = 181	N = 418	
Recipients			
Age, y	56 (48-64)	58 (50-64)	.54
Gender			.72
Male	133 (73.5%)	313 (74.9%)	
Female	48 (26.5%)	105 (25.1%)	
Race			.23
White	100 (55.2%)	238 (56.9%)	
Black	61 (33.7%)	110 (26.3%)	
Hispanic	14 (7.7%)	47 (11.2%)	
Asian	4 (2.2%)	12 (2.9%)	
Other	2 (1.1%)	11 (2.6%)	
Smoking history	66 (36.5%)	159 (38.0%)	.71
Diabetes mellitus	65 (35.9%)	134 (32.1%)	.36
Prior cardiac surgery	79 (43.6%)	174 (41.6%)	.65
BMI, kg/m^2^	29.7 (25.5-33.9)	28.2 (24.8-32.4)	.01
Functional status (1-10)	3 (2-7)	3 (2-6)	.61
Creatinine			<.001
≤1.5 mg/dL	109 (60.2%)	322 (77.0%)	
1.5-2.5 mg/dL	57 (31.5%)	81 (19.4%)	
≥2.5 mg/dL	15 (8.3%)	15 (3.6%)	
Total bilirubin, mg/dL	0.6 (0.4-0.9)	0.7 (0.5-1.2)	.02
Pulmonary hypertension	116 (64.1%)	263 (62.9%)	.79
Cardiac output, L/min	4.5 (3.71-5.61)	4.3 (3.56-5.25)	.08
Primary diagnosis			.07
Nonischemic dilated cardiomyopathy	108 (59.7%)	205 (49.0%)	
Ischemic cardiomyopathy	37 (20.4%)	123 (29.4%)	
Congenital heart defect	11 (6.1%)	14 (3.3%)	
Restrictive cardiomyopathy	10 (5.5%)	27 (6.5%)	
Valvular heart disease	0 (0.0%)	5 (1.2%)	
Hypertrophic cardiomyopathy	6 (3.3%)	19 (4.5%)	
Other	9 (5.0%)	25 (6.0%)	
Admission status			.02
ICU	107 (59.1%)	295 (70.6%)	
Hospitalized, non-ICU	36 (19.9%)	62 (14.8%)	
Not hospitalized	38 (21.0%)	61 (14.6%)	
Dialysis prior to transplant	14 (7.7%)	32 (7.7%)	.97
Mechanical ventilation	5 (2.8%)	22 (5.3%)	.18
LVAD	38 (21.0%)	68 (16.3%)	.16
tVAD	50 (27.6%)	118 (28.2%)	.88
IABP	28 (15.5%)	90 (21.5%)	.09
ECMO	22 (12.2%)	59 (14.1%)	.52
Status 1	40 (22.1%)	97 (23.2%)	.77
Status 2	89 (49.2%)	219 (52.4%)	.47
Status 3-6	52 (28.7%)	102 (24.4%)	.27
Days on waiting list	21 (11-60)	23 (8-69)	.69
Donors			
Age, y	37 (28-44)	34 (27-41)	.01
Gender			.85
Male	129 (71.3%)	301 (72.0%)	
Female	52 (28.7%)	117 (28.0%)	
Race			.89
White	109 (60.2%)	257 (61.5%)	
Black	31 (17.1%)	71 (17.0%)	
Hispanic	33 (18.2%)	73 (17.5%)	
Asian	3 (1.7%)	10 (2.4%)	
Other	5 (2.8%)	7 (1.7%)	
Smoking history	31 (17.1%)	57 (13.6%)	.27
Hypertension	33 (18.2%)	63 (15.1%)	.33
Cause of death			.25
Anoxia	80 (44.2%)	200 (47.8%)	
Cerebrovascular/stroke	23 (12.7%)	40 (9.6%)	
Head trauma	69 (38.1%)	164 (39.2%)	
CNS tumor	3 (1.7%)	1 (0.2%)	
Other	6 (3.3%)	13 (3.1%)	
BMI, kg/m^2^	28.3 (23.3-33.2)	26.7 (23.1-31.2)	.04
Creatinine, mg/dL	0.9 (0.7-1.7)	1.0 (0.7-1.6)	.90
Terminal pH <7.35	19 (16.2%)	37 (12.7%)	.34
Donor-recipient match			
Undersized donor heart	11 (6.1%)	20 (4.8%)	.29
HLA mismatch level	5 (4-5)	5 (4-5)	.65
Cross-clamp-to-cross-clamp time, h	4.0 (3.3-4.9)	3.8 (3.1-4.7)	.11

Categorical values are reported as frequency (% of population). Continuous values are reported as median (IQR). *BMI*, Body mass index; *CNS*, central nervous system; *ECMO*, extracorporeal membrane oxygenation; *HLA*, human leukocyte antigen; *HT*, heart transplantation; *IABP*, intra-aortic balloon pump; *LVAD*, left ventricular assist device; *tVAD*, temporary ventricular assist device.

**Table E7:** Causes of death for safety-net kidney-eligible recipients and isolated HT recipients who required postoperative dialysis

Causes of death	Safety-net kidney-eligible	Isolated HT with postoperative dialysis	*P* value
	N = 17	N = 89	
Cause of death			.23
Renal failure	0 (0%)	1 (1.1%)	
Multiorgan failure	0 (0%)	6 (6.7%)	
Graft failure	3 (17.6%)	5 (5.6%)	
Infection	1 (5.8%)	29 (32.6%)	
Cardiovascular	5 (29.4%)	20 (22.5%)	
Pulmonary	1 (5.8%)	6 (6.7%)	
Cerebrovascular	1 (5.8%)	6 (6.7%)	
Malignancy	1 (5.8%)	1 (1.1%)	
Other	3 (17.6%)	8 (9.0%)	
Unknown	2 (11.7%)	7 (7.9%)	

Data are presented as n (%). *HT*, Heart transplantation.

**Figure E1:**
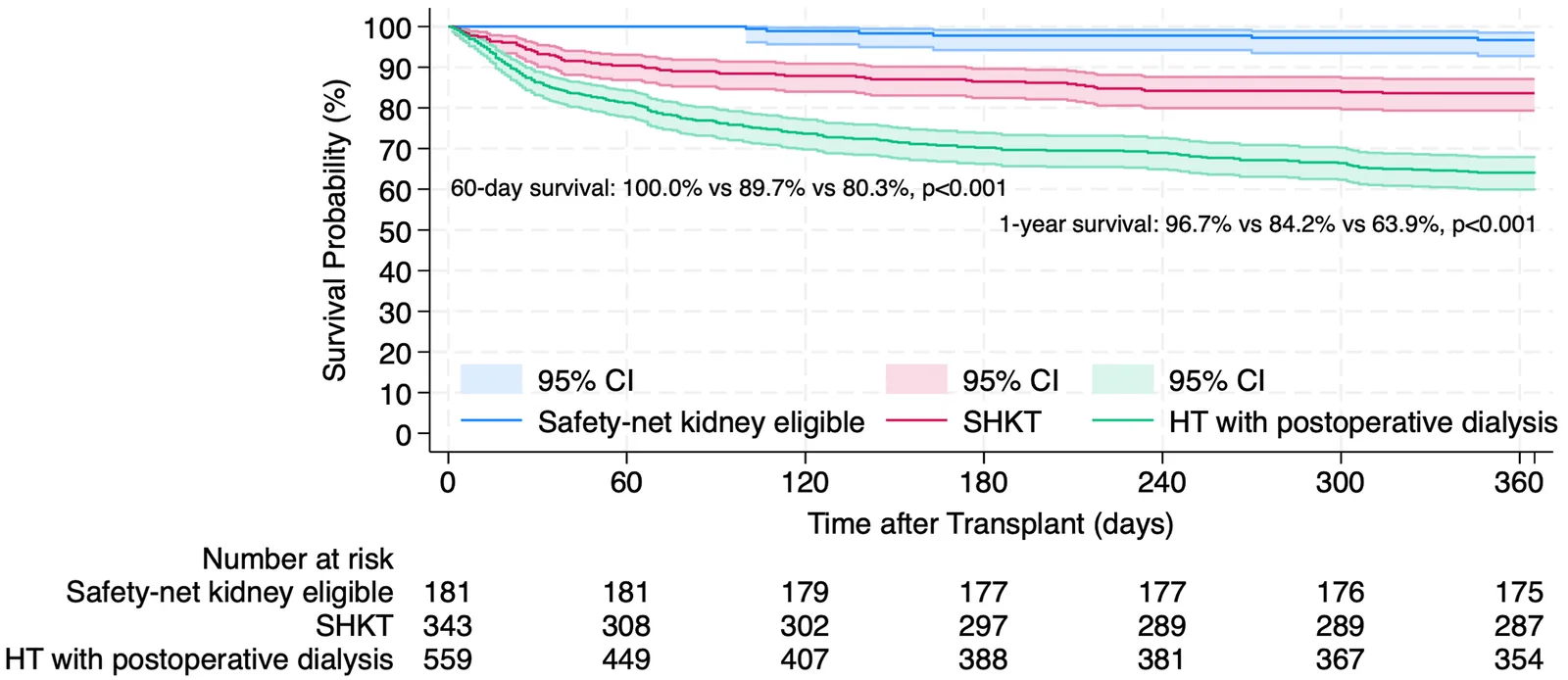
Uncensored 1-year Kaplan-Meier survival analysis of isolated HT recipients who required postoperative dialysis compared to safety-net kidney-eligible and SHKT recipients. *HT*, Heart transplant; *SHKT*, simultaneous heart-kidney transplant.
